# Adaptation of *Enterococcus faecalis* to intestinal mucus revealed by a human colonic organoid model

**DOI:** 10.1128/msystems.01304-25

**Published:** 2026-03-10

**Authors:** Sofya Mikhaleva, Po-Long Hsiao, Amanzhol Kurmashev, Caleb M. Anderson, Cristina Colomer-Winter, Julia A. Boos, Pei Yi Choo, Julia L. E. Willett, Andreas Hierlemann, Kimberly A. Kline, Alexandre Persat

**Affiliations:** 1 School of Life Sciences, Global Health Institute, École Polytechnique Fédérale de Lausanne (EPFL) 199935 , Lausanne, Switzerland; 2 School of Life Sciences, Institute of Bioengineering, École Polytechnique Fédérale de Lausanne 130376 , Lausanne, Switzerland; 3 Department of Biosystems Science and Engineering, ETH Zürich 211122 , Basel, Switzerland; 4 Department of Microbiology and Molecular Medicine, University of Geneva 218785, Geneva, Switzerland; 5 Singapore Centre for Environmental Life Sciences Engineering, Nanyang Technological University 54761, Singapore, Singapore; 6 Department of Microbiology & Immunology, University of Minnesota Medical School 12269, Minneapolis, Minnesota, USA; The University of Texas at Dallas, Richardson, Texas, USA

**Keywords:** *Enterococcus faecalis*, biofilm, mucus, organoid

## Abstract

The human gastrointestinal tract hosts a diverse population of microorganisms that have a significant impact on host health. Among this population, *Enterococcus faecalis* (*Ef*) represents a common member of intestinal microbiota, colonizing humans early in life but also capable of opportunistically infecting its host. Despite its importance in human health, investigations of its physiological adaptation to the mucosal environment remain limited. Building on recent advances in tissue engineering, we here leverage human colonic organoids (colonoids) to investigate the *Ef*’s mechanisms of mucosal surface colonization across space and time. Using high-resolution microscopy, we visualized *Ef* growth within the natively formed colonic mucus layer in colonoids. Leveraging a custom perfusion chamber, we tracked *Ef* growth within the mucus of live colonoids over time under flow, which revealed specific colonization strategies, including biofilm-like microcolony formation. To identify *Ef* fitness determinants in this niche, we implemented transposon insertion sequencing (Tn-seq) in the natively formed mucus of live colonoids. This approach revealed a large fitness rearrangement compared to typical liquid culture, mainly involving metabolic activity and regulatory response during mucosal colonization, as well as factors that may contribute to colony formation at the mucosal surface. Altogether, our results show important physiological and biophysical adaptation of *Ef* to the mucosal surface that are not captured by *in vitro* conditions and that cannot be revealed *in vivo* at high resolution.

**IMPORTANCE:**

Gut microbiota interactions with mucus during early intestinal colonization are critical for establishing stable communities and influencing host health. Using human colonic organoids combined with Tn-seq and live imaging, this study reveals how *Enterococcus faecalis* adapts to the mucosal surface by forming microcolonies and reprogramming its metabolism. This integrative approach provides a powerful platform to study other microbiota members in the native-like environment of the large intestine and evaluate potential therapeutic interventions.

## INTRODUCTION

The human gastrointestinal (GI) tract hosts communities of bacterial species collectively known as the gut microbiota. Many members play key roles in digesting complex dietary fibers, strengthening gut barrier integrity, and shaping the host immune system (1, 2). Despite these important functions, the mechanisms by which any given bacterial species stably colonizes the gut remain poorly characterized, in part due to our limited ability to monitor host colonization in space and time at high resolution. The large intestine hosts a particularly abundant bacterial population, physically separated from the colonic epithelium by a thick mucus layer. Mucus is a hydrogel secreted by dedicated goblet cells, predominantly built from the crosslinked chains of heavily glycosylated MUC2 mucin proteins. While the main function of mucus is to physically repel microbes from host epithelial cells, it also acts as a nutrient source to some bacterial species. Many species can degrade mucus and release sugars to nourish the rest of the microbiota (3, 4). As mucus is a complex, heterogeneous material, meticulously investigating its dual function remains complex in animal models, which face limitations in live, dynamic visualizations. Conversely, *in vitro* models building on purified mucus often fail to recapitulate its physical integrity upon purification. Consequently, our understanding of colonization and stability of pathogens and commensal microbiota at the mucosal surface is still limited (5–7).

*Enterococcus faecalis* (*Ef*), a Gram-positive non-motile facultative aerobe, is a core member of the human intestinal microbiota (8, 9). As a highly adaptable commensal and one of the earliest GI tract colonizers (10–12), *Ef* supports intestinal physiology by regulating pH, facilitating metabolism, and producing vitamins (13). However, under certain perturbations, including antibiotic-induced dysbiosis or inflammatory bowel disease, *Ef* can become an opportunistic pathogen breaching the epithelial barrier, causing serious infections like bacteremia and endocarditis (14–18). These pathogenic traits are linked to exotoxin production, antibiotic or bile acids tolerance, and biofilm formation (19, 20). *Ef* may also employ physical strategies to persist in the host. It specifically grows into multicellular mucus-associated structures that resemble biofilms, thus likely offering protection from flow-induced clearance (5). Despite *Ef*’s role in early GI tract colonization and pathogenicity, most existing knowledge of *Ef* biofilms derives from studies conducted in abiotic environments. These studies have revealed that sortase-regulated adhesins mediate initial surface attachment (21), while secretion of polysaccharides (22, 23) and extracellular DNA (24) promotes biofilm maturation and cohesion. Commitment to form biofilms by any species has a strong impact on bacterial interaction with its environments, for example, by restricting nutrient access, which can induce metabolic reprogramming (25, 26). The way in which *Ef* and other microbiota species adapt their physiology to form biofilms at mucosal surfaces remains largely unexplored (5, 27).

To elucidate the mechanisms of bacterial colonization in a more physiologically relevant context, recent advances in tissue and organoid engineering offer a promising alternative. Human-derived organoids bypass limitations of animal experiments, which do not always reflect human physiology. In addition, careful organoid engineering enables high-resolution microscopy and functional genomics integration. For example, we have previously demonstrated that human airway organoids can highlight *Pseudomonas aeruginosa* mechanisms of biofilm formation and pathogenic adaptation during growth and antibiotic treatment (28, 29). Intestinal organoids have also been employed to investigate enterohemorrhagic *Escherichia coli* (30)*, Salmonella* (31)*, Clostridioides difficile* (32)*,* and *Shigella flexneri* infections of the GI tract (33), as well as *Vibrio cholerae* biofilm formation on immune cells after breaking intestinal barriers (34).

To investigate *Ef* adaptation strategies to the gut mucosal barrier in space and time, we developed a framework combining mucus-producing tissues from human colonic organoids (colonoids), live imaging, and a functional genomic screen. Fluorescent microscopy revealed that *Ef* grows in mucus-rich regions by forming dense microcolonies, akin to those observed in mouse and human intestines (12, 35). Using transposon sequencing (Tn-seq), we identified key factors involved in *Ef*’s metabolic and physical adaptation to the gut mucosal environment. While several known biofilm-associated factors in our Tn-seq results were dispensable during early colonization, the glycosyltransferase BgsB emerged as critical for *Ef*’s initial adaptation to mucus. We propose that this combined platform of human colonoids, live imaging, and functional genomics offers a broadly applicable tool to study adaptation and pathogenicity in intestinal pathogens, commensals, and microbial communities.

## RESULTS

### *Ef* colonizes colonoid mucus

**Fig 1:**
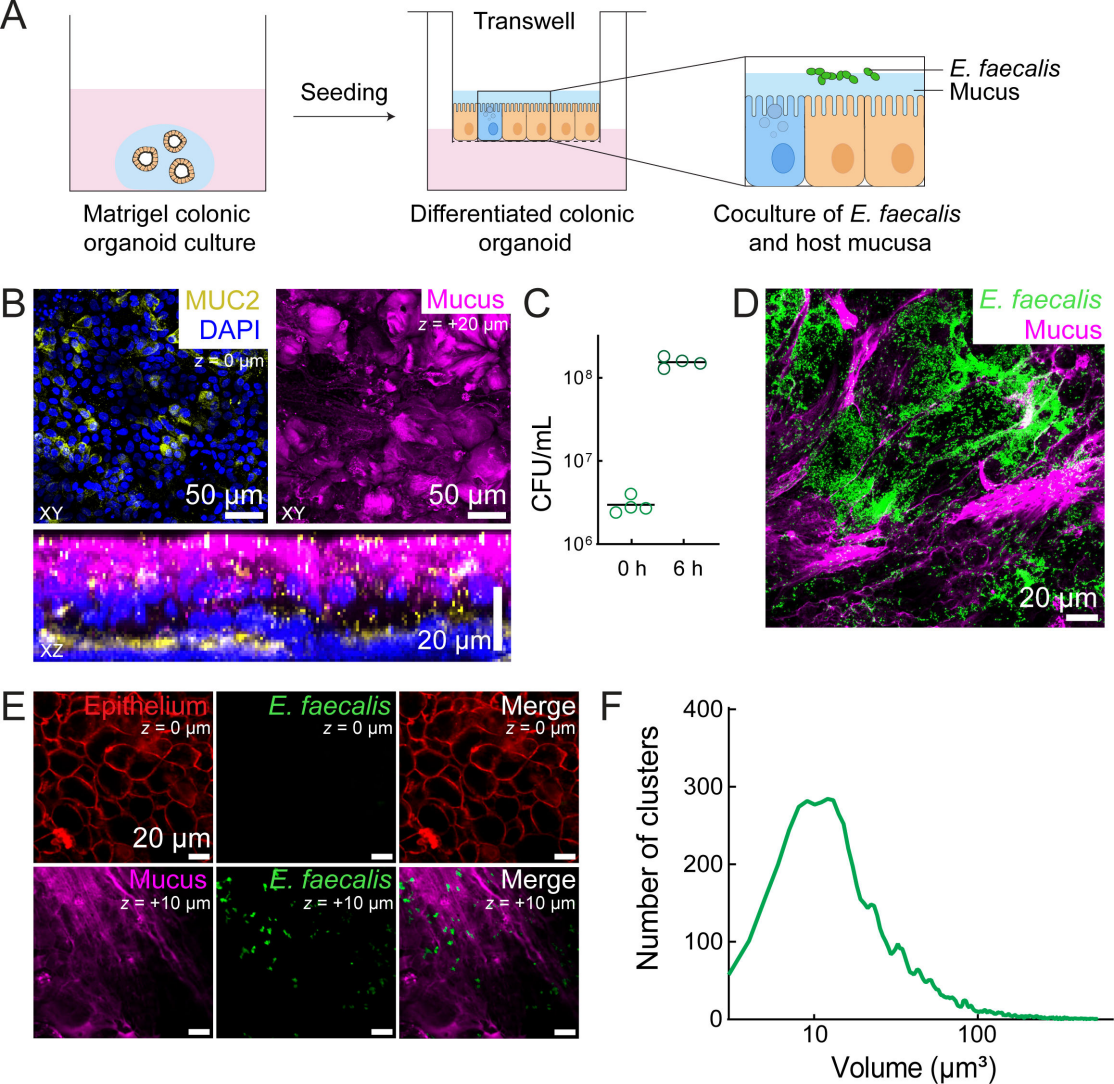
Colon epithelial monolayers with histological signatures for studying *Enterococcus faecalis* mucosal colonization. (**A**) Schematic depicting the experimental setup. Human colon organoids grown in Matrigel were split into single cells and seeded on top of collagen-coated Transwell membranes. Once the epithelial monolayer formed, the cells were differentiated at the air-liquid interface. Transwells were used on day 7 after seeding. (**B**) Immunostaining images of differentiated organoid monolayers with MUC2 protein (yellow), nuclei stained with DAPI (blue), and mucus labeled using Jacalin-biotin and streptavidin-Cy5 (magenta). (**C**) Colony-forming units (CFU/mL) analysis of *Ef* growth in mucus 6 h after inoculation. Both time points represent the colony-forming units of bacteria present in the Transwell in four independent biological replicates. The horizontal black lines mark the mean. (**D**) Maximum intensity projection of Jacalin-labeled mucus (magenta) and *Ef* WT expressing pDasherGFP (green). (**E**) *Ef* WT expressing pDasherGFP (green) in the same z-plane as the Jacalin-labeled mucus (magenta) and does not colocalize with the colonoid epithelium labeled with CellMask-DeepRed (red). (**F**) *Ef* colony volume calculated from confocal images represented in panel **C**. A minimum of 148 clusters was quantified from each image of four independent biological replicates.

To investigate how *Ef* thrives in a human mucosal environment, we use patient-derived healthy descending colon organoids. Widely used for developmental studies, organoids typically grow in a cystic shape, wherein the epithelial lumen faces inwards (36). Colonization and infection thus require microinjection to access the mucosal surface, thereby undermining organoid integrity (37). To overcome this limitation, we leveraged the open apical configuration of organoids grown on a 2D membrane (Transwell) (38, 39), forming a polarized epithelium with the apical side facing upward. This allows us to directly access the mucosal surface for microbial inoculation without perturbing the tissue (Fig. 1A).

Differentiated colonoids with mucus-secreting goblet cells were shown by immunostaining with anti-MUC2 antibody (Fig. 1B). To produce an *in vivo*-like mucus quantity, we enhanced mucin production by supplementing the culture with vasoactive intestinal peptide (VIP) (39), resulting in its visible accumulation. To confirm the presence of gel-forming mucins at the epithelial surface, fixed colonoids were labeled with a fluorescent conjugate of the lectin Jacalin, which binds human colonic MUC2 O-glycans (40, 41). Despite extensive washing during fixation and staining steps, which erode mucosal content, Jacalin-staining revealed a ~10 µm thick extracellular mucus layer (Fig. 1B), mimicking the native colonic environment. We therefore used this platform to interrogate *Ef’s* physiology in intestinal mucus.

We first tested the ability of *Ef* to grow at the mucosal surface of 2D colonoids. In this configuration, organoids are exposed to media through the porous membrane at the basal side, while we maintained an air-liquid interface at the apical side, which promotes mucus accumulation. This setup allowed us to assess *Ef’s* adaptation to the mucosal surface, where bacteria experience mucus without exogenous nutrients. To prevent nutrient carryover from prior cultures, *Ef* was thoroughly washed and inoculated in a salt solution in small volumes that preserved the air-liquid interface. To monitor bacterial growth, we quantified colony-forming units (CFU) per Transwell and compared them to the initial inoculum. After 6 h, the number of viable *Ef* cells increased approximately 50-fold, indicating favorable growth conditions (Fig. 1C). This corresponds to a doubling time of ~1 h, which is slower than in the brain-heart infusion (BHI) medium (42), suggesting that *Ef* experiences nutrient-limited conditions. Altogether, these experiments suggest that *Ef* could utilize host-derived nutrients, such as mucin glycans and amino acids, to grow at the mucosal surface.

Next, we investigated the patterns by which *Ef* populations colonize the mucosal surface of colonoids. To visualize the spatial organization of *Ef* around mucus, we leveraged the optical accessibility of human colonoid tissues. We colonized colonoids with an *Ef* strain constitutively expressing GFP (43), and the mucus was stained with Jacalin-biotin and streptavidin-Cy5 before imaging the tissues with 3D confocal spinning disk microscopy (Fig. 1D). We could observe that *Ef* preferentially formed microcolonies in mucus-rich areas, where it remained separated from epithelial cells (Fig. 1E). Cluster size analysis revealed a mean volume of ~28 µm³ (Fig. 1F). Assuming the cluster grew from a single 0.5 µm³ bacterial cell (1 µm diameter sphere), volume expansion approached 50-fold, consistent with CFU measurements (Fig. 1C). These observations together suggest that *Ef* not only survives but proliferates at the colonoid mucosal surface without external nutrients, forming large microcolonies reminiscent of biofilms. We therefore hypothesize that *Ef* exploits mucus-derived nutrients to sustain growth and establishes a stable, host-adapted lifestyle at the mucosal surface.

### *Ef* forms multicellular clusters embedded in mucus

**Fig 2:**
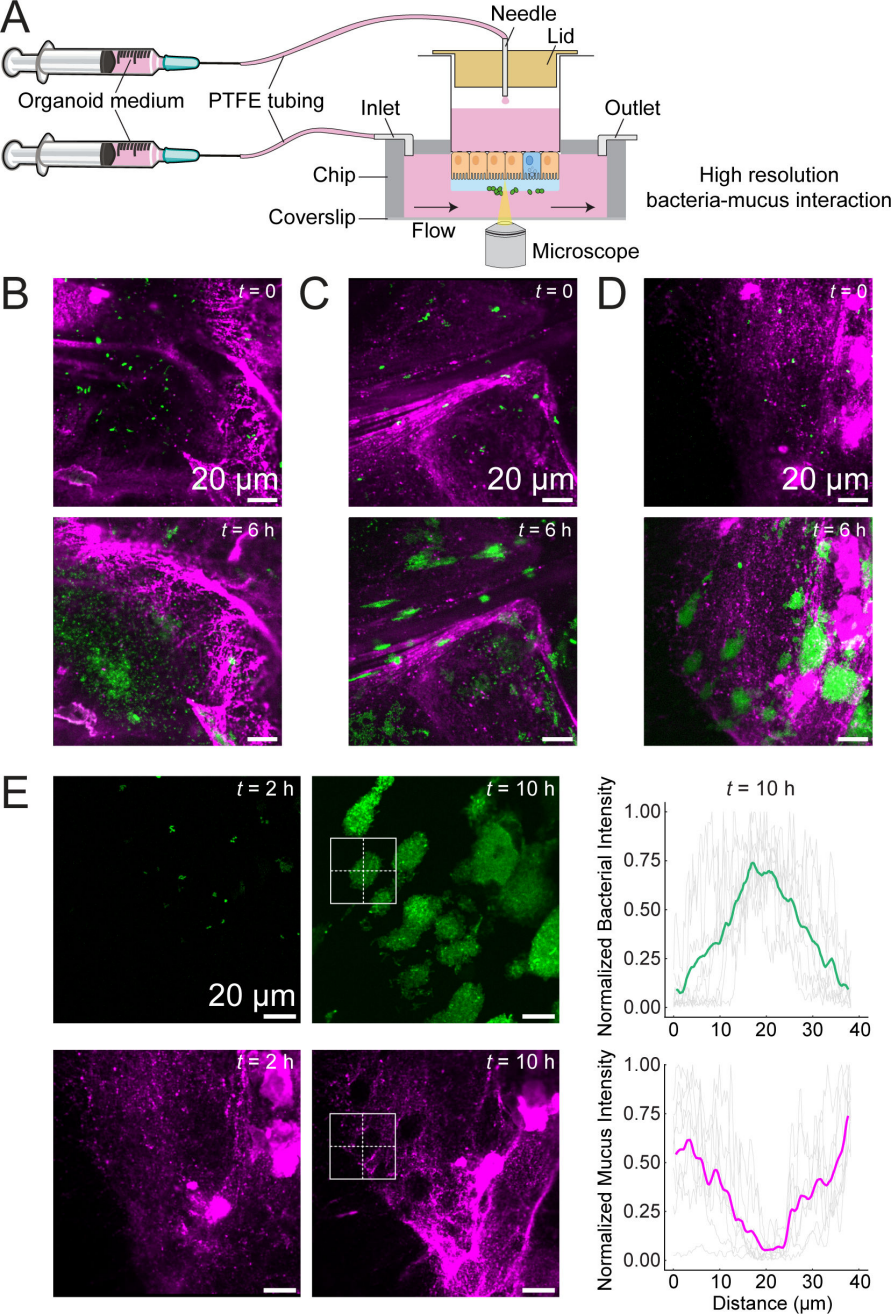
*Ef* grows in cavities surrounded by mucus. (**A**) Schematic depicting the experimental setup using a Transwell-adapted microfluidic device for high-resolution confocal imaging. (**B**) *Ef* colonies growing between thick mucus fibers and not forming biofilm-like colonies (*n* = 2). (**C**) *Ef* colonies growing inside the mucus but in areas with low mucus labeling (*n* = 3). (**D**) *Ef* colonies growing and slowly pushing Jacalin-labeled mucus away upon expansion. (**E**) Images of the bacterial channel (green) and mucus channel (magenta) of the time-lapse confocal imaging of *Ef* in mucus under flow. Graphs represent the mean of quantification of the sum of the vertical and horizontal profile measurements (dashed lines within the cropped region) of nine colonies analyzed in the bacterial channel (upper right) and mucus channel (lower right).

To investigate the morphogenesis of *Ef* biofilms at the mucosal surface, we performed time-lapse microscopy under flow that replicates the conditions experienced in the GI tract. To enable the application of controlled flow, we first grew colonoids at the bottom side of the Transwell membrane rather than the top, in a so-called inverted configuration. Once the organoids have differentiated, we then used a custom microfabricated microscopy adaptor that precisely positions the tissue in close proximity to the coverslip for high-resolution imaging (44). The device also incorporates channels with inlets and outlets, enabling steady fluid perfusion (Fig. 2A). In addition to replicating intestinal flow, this setup may also remove some detached bacterial biomass, enabling accurate tracking of mucus-bound populations. We pre-labeled mucus with Jacalin-biotin and streptavidin-Cy5 before inoculating GFP-expressing *Ef* directly onto the mucus. We used a flow intensity on the same order as the one experienced in the large intestine (45). This setup allowed us to perform live microscopic tracking of bacterial growth at the mucus-medium interface. We first examined *Ef* growth over time at defined regions of the mucosal surface. *Ef* grew into groups of different densities, consistently localized away from epithelial cells but always near mucus-rich regions. Visualizing *Ef* at single-cell resolution revealed heterogeneity in growth behaviors. We performed experiments in organoid medium with high glucose content to speed up growth (Fig. 2B through E).

Bacteria tended to adopt one of two distinct lifestyles: low-density communities within large spaces formed between mucus strands (Fig. 2B) or densely packed clusters of contiguous *Ef* cells confined within narrow mucus-lined trenches (Fig. 2C). Among those, we observed large bacterial clusters formed within cavities surrounded by mucus that were likely created by the expansion of the colonies (Fig. 2D and E). To test this, we performed experiments of colonoid colonization in a minimal medium where growth was limited. These showed attachment of small aggregates which did not generate cavities in mucus, indicating that the cavities in Fig. 2E are formed by strong growth of biofilm-like colonies (Fig. S1B and C). Overall, we observed limited overlap between *Ef* and Jacalin-labeled mucus. Fluorescence intensity quantification confirmed that most clusters grew in areas of low mucus fluorescence, surrounded by a strong mucus signal (Fig. 2E), suggesting that the elastic, gel-like structure of mucus supports and actively shapes *Ef* growth. Together, these findings show that under flow, *Ef* preferentially colonizes and grows within Jacalin-labeled mucus cavities, rather than at the surface.

### Transposon sequencing in colonoids reveals *Ef* mucus adaptation strategies

Our imaging data indicate that *Ef* rapidly adapts to the mucosal surface, growing using host-derived nutrients. Its slower growth rate in mucus (Fig. 1C) compared to that in the BHI broth (42) suggests underlying metabolic reprogramming and specialized phenotypes required for mucus adaptation. We hypothesized that these adaptive strategies differ significantly from those used in rich medium cultures. To identify genetic factors contributing to growth on mucus, we conducted a high-throughput functional genomic screen in colonoids. We performed transposon sequencing (Tn-seq) using a *Himar*-based transposon library of the OG1RF strain with 1,926 insertions of 2,651 total annotated open reading frames (46). We anticipated that mutants in genes important for adaptation to mucus would show reduced fitness.

**Fig 3:**
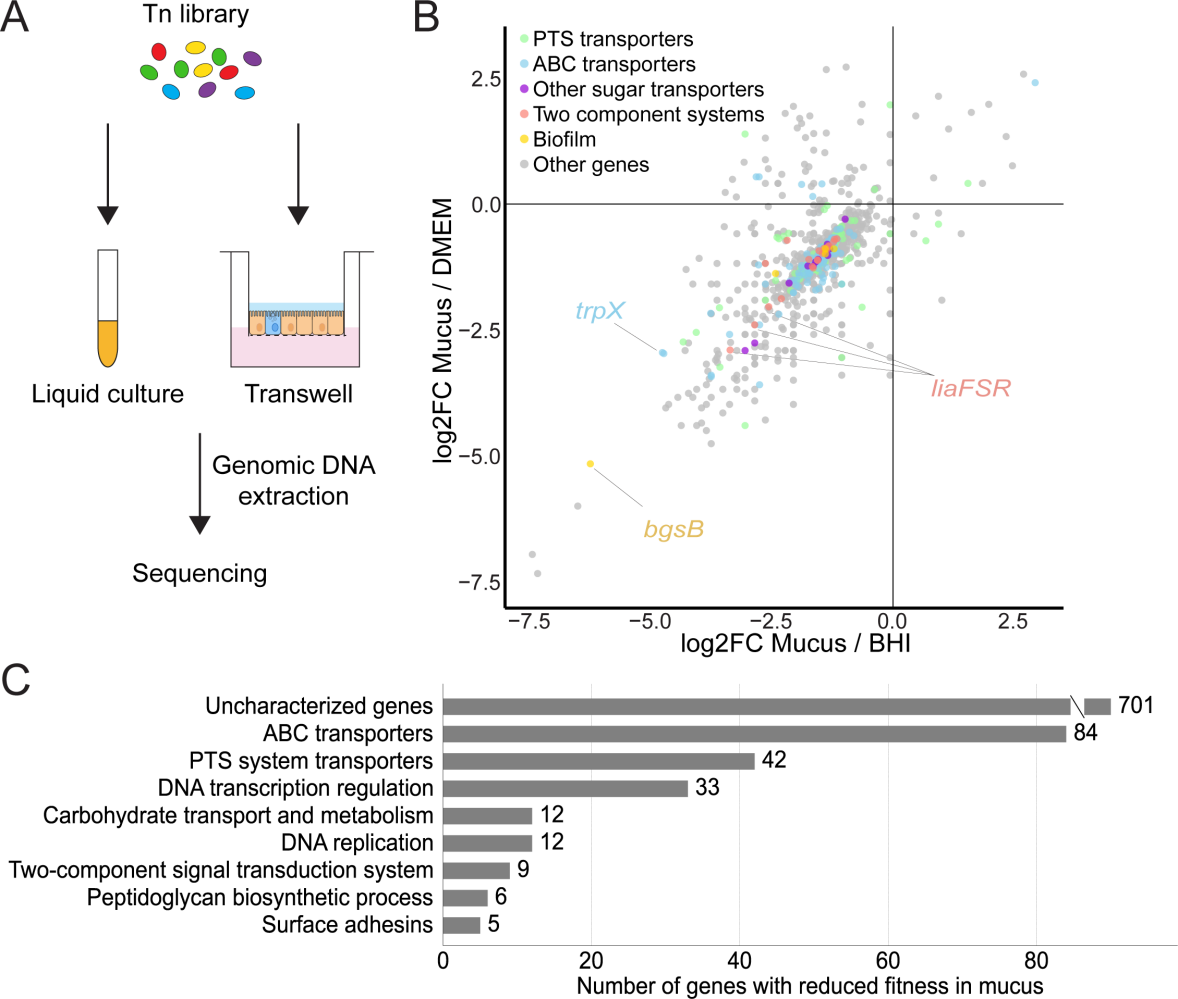
Tn-seq in colonoids reveals mechanisms of adaptation to the mucosal surface. (**A**) Scheme depicting the experimental setup for comparing *Ef* growth in colonoids vs liquid media. (**B**) Scatter plot representing the distribution of transposon mutants in mucus with highlighted carbohydrate transporters in blue, biofilm-related genes in yellow, and two-component signal transduction adaptation system genes in red. Genes with the largest fold change in reduced fitness in these categories were highlighted. (**C**) Biological processes found in a gene ontology analysis of the Tn-seq results (KEGG pathway).

We cultured the Tn library and compared its fitness in the colonic mucus conditions to growth in the standard BHI medium, which was used to grow the library prior to inoculation. To control interference from organoid medium leakage through the epithelium, we sequenced a second reference condition with the library growing in the Basal medium used in colonoid tissue culture (Fig. 3A). Incubation times were limited to six division cycles (Fig. S2A), during which *Ef* forms large colonies in the mucus layer but does not cause any large-scale tissue damage, as observed by propidium iodide staining (Fig. S2B). Under these conditions, we expected that selection would primarily reflect the inability of mutants to grow in mucus, rather than effects resulting from epithelial damage.

Analysis of the Tn-seq identified 569 genes with reduced fitness (log2FC < −2) in mucus compared to growth in BHI, many of which were annotated as hypothetical (Table S1). Among genes of known function, we identified genes responsible for sugar uptake, including ABC and PTS system transporters and carbohydrate transporters, indicating adaptation to distinct nutrient conditions and consistent with the reliance on host-derived glycans. Furthermore, mutations in genes associated with environmental adaptation, such as two-component signal transduction systems, suggest that *Ef* modulates its physiology in response to specific inputs at the mucosal surface. Finally, mutations in biofilm factors, such as peptidoglycan biosynthesis and surface adhesins, indicate that the biofilm lifestyle may promote fitness in the mucosal environment (Fig. 3B and C).

### *Ef* metabolically adapts to mucus

Tn-seq in mucus identified metabolic and two-component system genes to be important in mucosal adaptation (Fig. 3C). Mucins, the primary glycoproteins in mucus, are heavily glycosylated and contain a variety of sugar residues in their O- and N-glycans, including mannose, fucose, galactose, N-acetylglucosamine (GlcNAc), N-acetylgalactosamine (GalNAc), and sialic acids (3, 4). Our Tn-seq screen showed strong fitness defects for mutations in genes involved in tryptophan and methionine biosynthesis, as well as an operon of galactose/GalNAc phosphotransferase transporters (Fig. S3A). We validated this observation and observed that mutations in *trpX* and *metQ* genes resulted in ~30% reduced growth rate compared to WT (Fig. S3B).

Additionally, we identified two previously uncharacterized genes of the ABC family sugar transporters that are important for *Ef* growth on mucus (Fig. S3A). We found that mutations in *OG1RF_10303* and *OG1RF_12205* result in a significant fitness reduction in mucus (Fig. S3B). Consistent with our Tn-seq results, we found that these mutants had no growth defects in rich medium. To identify the mechanisms by which they help *Ef* metabolically adapt at the mucosal surface, we screened the growth of these mutants in a minimal medium supplemented with sugar residues that can be released from mucus. We found that a mutant in *OG1RF_11614* results in a strong fitness defect in GalNAc, while the growth of *OG1RF_10303* and *OF1RF_12205* mutants was unaffected by the sugars we could screen (Fig. S3C). These data confirm that *Ef* deploys metabolic transporters compatible with nutrient availability at the mucosal surface.

Genes coding for the glycosyl hydrolase families, such as GH18 and GH20 N-acetylglucosaminidases, GH2 and GH35 galactosidases, and GH101 N-acetylgalactosaminidases, are often associated with mucin degradation (47, 48). While they did not pass the significance threshold in our analysis, we still compiled the fitness of mutants in the genes coding for those carbohydrate-active enzymes in Table S1, selected using the CAZy Database for the OG1RF strain (49). In addition, the functional genomics screen identified three two-component sensory systems (the *Ef* OG1RF genome encodes 16) that may play a role in mucosal adaptation (Fig. S4A). Mutations in the *croRS*, *liaFSR,* and *bsrRS* two-component systems decreased fitness. We validated the reduced fitness of the *bsrRS* mutant in colonoids by CFU analysis, confirming that *Ef* rapidly senses specific signals to adapt to the intestinal mucosal environment (Fig. S4B). Which signals activate the system at the mucosal surface, however, remains unclear.

### BgsB is essential to multicellular clusters formation in mucus

**Fig 4:**
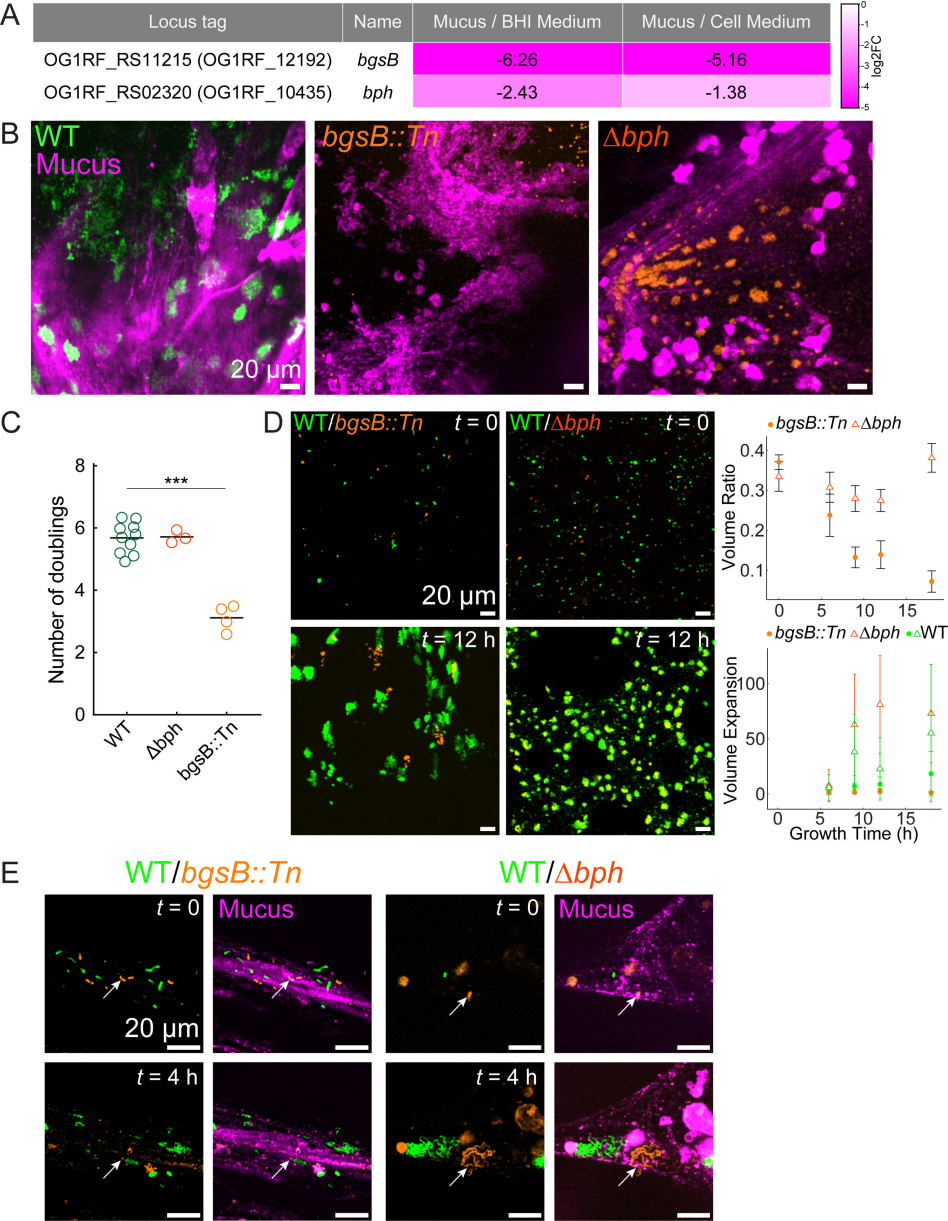
Glycosyltransferase BgsB in *E. faecalis* is essential for growth in colonic mucus. (**A**) Table of log2FC values calculated from the Tn-seq experiment for the glycosyltransferase *bgsB* and a biofilm-forming factor *bph*. (**B**) Representative images of *Ef* colonies in colonic mucus at 9 h of growth from three biological replicates. Mucus was labeled using Jacalin-biotin and streptavidin-Cy5. Wild-type *Ef* fluorescently expressed pDasher-GFP, and *bgsB* and *bph* mutants expressed tdTomato. (**C**) Colony-forming unit (CFU/mL) quantification of the deletion mutants grown in mucus compared with the wild-type *Ef* after 6 h of growth. Each dot is a biological replicate (at least three independent biological replicates for each mutant), and the horizontal black lines mark the mean. (**D**) Competition between WT (green) and mutant samples (orange) with over three biological replicates, where the WT vs *bgsB::Tn* is marked with a filled circle and WT vs Δ*bph* with an unfilled triangle. Volume ratio is calculated as a fraction of the mutant signal occupying the total (mutant + WT) volume in the image. Volume expansion is calculated as a ratio to the initial volume in the respective channel. Mean and standard deviation are shown. (**E**) The same mixed samples were grown in mucus in a flow chip shown in Fig. S1A, under the flow of 5 µL/min of minimal medium (*n* = 3). Arrows indicate the same place at the start and the end of the time-lapse. Statistics in panel **C** were calculated using one-way ANOVA with post hoc Dunnett’s multiple comparison test (***P < 0.001).

Given the formation of large biofilm-like colonies in mucus, we investigated whether interfering with this process could impact *Ef* fitness. We mined the Tn-seq data for genes linked to biofilm formation on abiotic surfaces and identified two that exhibited significant fitness defects when grown in mucus (Fig. 4A). One was *bph*, a biofilm-forming factor whose deletion decreases the expression of the *fsr* locus, gelatinase (*gelE*), and multiple cell surface WxL domain proteins, all of which contribute to *in vitro* biofilm formation and surface attachment (50, 51). Another candidate was *bgsB*, a glycosyltransferase required for diglucosyldiacylglycerol synthesis and biofilm formation (52).

We confirmed that the *bgsB::Tn* mutant exhibited significantly reduced fitness in mucus compared to WT (Fig. 4B and C). By contrast, CFU did not confirm a fitness defect for the *bph* deletion mutant. Growing in minimal medium conditions, the Δ*bph* mutant showed a delayed logarithmic growth when mannose was the only carbon source compared to WT (Fig. S5). To assess how *bgsB* impacts *Ef* mucosal colonization, we returned to imaging. In the regular Transwell setting (shown in Fig. 1A), confocal microscopy showed that the *bgsB::Tn* mutant failed to form biofilm-like microcolonies, while the Δ*bph* mutant formed WT-like colonies both in monocultures and co-culture with WT at a 1:1 ratio (Fig. 4B through D). High-resolution imaging of *Ef* growth with mucus fibers using the Transwell-based microfluidic platform (shown in Fig. S1A) confirmed the growth defect of the *bgsB::Tn* mutant and showed similar growth for WT and the Δ*bph* mutant (Fig. 4E).

## DISCUSSION

Despite extensive *in vitro* and *in vivo* investigation of gut microbial species, we still lack a clear understanding of bacterial physiology at the mucosal surface. Our work shows that human colonic organoids represent a suitable model system to bridge mechanistic studies with animal or human data. We employed colonoids for live visualization of *Ef* colonization dynamics within a physiologically relevant human mucosal environment, thereby revealing both metabolic and biophysical adaptation strategies. By combining human colonoid-derived tissues, live imaging, and Tn-seq screening, we directly observed *Ef* microcolony formation and identified genetic determinants of fitness under the nutrient-limited and physically complex conditions of the mucus. This allowed us to focus on the early colonization and adaptation events that *Ef* undergoes when transitioning from a liquid culture to a native-like intestinal mucus environment. This process could be similar to the one initially employed by *Ef* to colonize the infant gut, where *Enterococcus* and *Escherichia* species are among the early pioneers, thereby forming a favorable niche for subsequent anaerobic species colonization (53). Our findings show that *Ef* strongly prefers the mucosal niche, rapidly attaches, and forms robust, biofilm-like colonies, as previously reported in both germ-free mouse models and patient-derived intestinal samples (12, 35). These results deepen our understanding of how a typical commensal exploits host-derived nutrients and stress-sensing pathways to adapt and stably populate on the mucosa, bridging a key gap between traditional *in vitro* biofilm assays and *in vivo* colonization models.

By growing human intestinal tissue on the basal side of the Transwell membrane, we were able to observe bacteria–mucus interactions in real time, capturing the entire process from the initial binding of a single bacterial cell to the formation of a biofilm-like, multicellular structure (Fig. 2; Fig. S1). We observed *Ef* colonies associated with mucus, either distributed between mucus fibers (Fig. 2B and C; Fig. S1B and C) or embedded within the bulk of the gel matrix, which was reshaped during growth (Fig. 2D and E). Given that *Ef* can degrade mucus aerobically in a glucose-independent manner (54), we postulate that *Ef* may be able not only to degrade but also mechanically displace the mucus matrix. We obtained these results using a simple yet robust Transwell-based microfluidic platform that supports high-resolution live-cell imaging (44). Such dynamic colonization events are typically obscured in animal models, and to our knowledge, this represents the first real-time microscopic observation of bacterial commensal biofilm-like colony formation in native human intestinal mucus.

Our Tn‐seq screen further highlighted the predominance of metabolic flexibility and stress‐response systems in early mucosal adaptation. Genes involved in ABC/PTS carbohydrate transporters and amino acid biosynthesis (methionine and tryptophan) were essential for growth on mucus, reflecting the reliance on host‐released glycans and the scarcity of free nutrients in the mucosal environment. Particularly, since methionine and tryptophan are essential amino acids for *Ef* (55) and are neither abundant in the mucus nor secreted by the host (56), *de novo* synthesis becomes essential for survival in this niche. Although not specifically tested for, our data has also suggested that some glycosyl hydrolases may be important for the metabolic adaptation of *Ef* to the mucosal niche. We expected that mucus-associated enzymatic activity of members of GH18 (endo-β-N-acetylglucosaminidase OG1RF_12167 and chitinase OG1RF_10250) and GH20 (bifunctional endo- and exo-β-N-acetylglucosaminidase OG1RF_10107) families, which can specifically cleave N-linked glycans on mucosal glycoproteins (57–59), would play a role in our screen, but they did not pass the significance threshold (Table S1). This might be due to the short timescale of our experiments, but their function might play a role during long-term colonization and should be further investigated. Additionally, enzymes with β-galactosidase activity, encompassing OG1RF_10545 (GH38), OG1RF_11514 (GH35), and OG1RF_12076 (GH2), as well as endo-α-N-acetylgalactosaminidase OG1RF_11509 (GH101), may be involved in mucin degradation. Also, the two‐component envelope stress-sensing system BsrRS (analog of BsrXRS in *Enterococcus faecium* [60]) emerged from our Tn-seq analysis, suggesting that *Ef* must rapidly detect and respond to chemical stressors encountered at the mucosa. Collectively, these data portray *Ef* as a metabolically versatile and environmentally responsive opportunist, capable of fine-tuning its physiology to colonize the colonic mucosa.

To better understand the molecular mechanisms underlying the microcolonies observed by microscopy, we compared our Tn-seq results with known biofilm-associated genes. We found that key biofilm-associated factors, including Fsr quorum-sensing system or sortase-regulated adhesins (50, 51), while important in abiotic settings and in the *in vivo* gut colonization model (21), were dispensable for early mucosal colonization (Table S1). This suggests that the demands of colonizing the mucus environment differ significantly from those of classical *in vitro* biofilm models. Notably, virulence factors associated with the biofilm formation and pathogenicity of *Ef*, such as the Fsr-regulated enzymes GelE and SprE (51), were both non-significant in our Tn-seq results (Table S1). This is consistent with commensal-like behavior in the mucus. Another notable *epa* gene cluster, which is involved in polysaccharide biosynthesis, has previously been shown to play a role in normal cell growth and division, pathogenesis, and *in vitro* biofilm formation (51, 61). Our data indicate that the *epa* cluster, particularly the glycosyltransferase genes OG1RF_11724 (EpaO), OG1RF_11725 (EpaN), and OG1RF_11714/11715 (EpaOX), might be important for mucus colonization and microcolony formation (Table S1). Additionally, this multicellular lifestyle, which mimics native biofilm-like structures observed in intestinal tissue, may exploit the mucus matrix itself for protection against flow-mediated clearance in the absence of other species. Notably, without external stress, such as antibiotics or competing microorganisms, biofilm formation appears not essential for stable colonization in mucus.

One of the factors we identified as essential for early mucus colonization is the glycosyltransferase BgsB. Mutations of *bgsB* abrogated microcolony formation and fitness in our gut organoid model, highlighting the importance of cell membrane composition and associated glycolipids for the attachment and growth of *Ef* in clusters within mucus. Future studies will investigate how BgsB-mediated modifications influence host–microbe interactions and whether targeting this pathway could selectively prevent *Ef*’s pathogenic overgrowth. By sequencing the genetic library and imaging colony growth over short periods, we were able to avoid the point at which *Ef* would typically overgrow and infect the tissue. However, the underlying mechanisms that drive the transition of *Ef* from a commensal to a pathogen in the gut, shifting from homeostasis to dysbiosis, remain unresolved. Identifying the factors that influence this transition will be an important next step for this model, which could also facilitate direct drug testing and the examination of polymicrobial communities within the same experimental setup.

Organoids are now becoming indispensable tools for dissecting host–microbe interactions in conditions that most resemble the human mucosal surface, offering an intermediate complexity between oversimplified *in vitro* assays and overly complex, not always relevant, animal models. Human colonoids recapitulate key features of the large intestine, such as polarized epithelium, mucin-secreting goblet cells, and a native mucus layer. These elements are absent in conventional carcinoma-derived cell lines, yet they are critical for shaping the native-like host environment and investigating microbial colonization in this context. One critical future direction is to develop organoid systems capable of generating double-layered mucus (62) and an anaerobic environment (63), which better mimic the native colonic barrier and enable more accurate modeling of host–microbe interactions. In this study, we showed that colonoids uniquely capture the dual biophysical-metabolic interface of the gut mucus niche, making them particularly well suited for studying commensal stability, dysbiosis, and pathogen emergence within the microbiome (64).

## MATERIALS AND METHODS

### Bacterial culture conditions

Bacteria were cultured in BHI medium (Sigma Aldrich cat. no. 53286-500G), with all liquid cultures incubated at 37°C without shaking unless otherwise indicated. Strains used can be found in Table 1. Antibiotics were used with overnight cultures of fluorescent strains. Spectinomycin (Chemie Brunschwig cat. no. J61820-06) was dissolved in water at 60 mg/mL and stored at −20°C until use at a working concentration of 120 µg/mL.

**TABLE 1:** Reagents and strains

Reagent or resource	Source	Identifier
Antibodies and lectin staining		
Purified mouse anti-human MUC2	BD Pharmingen	#555926
Goat anti-mouse IgG (H+L) cross-adsorbed secondary antibody, Alexa Fluor 594	Life Technologies	A11005
Jacalin-biotin	Vector Laboratories	VC-B-1155-M005
Streptavidin-Cy5	Vector Laboratories	SA-1500-1
Strains		
OG1RF Tn-seq library	(61)	
OG1RF WT	(65, 66)	
OG1RF WT pDasher-GFP	(43)	
OG1RF *bgsB::Tn*	(46)	
OG1RF Δ*bph*	(61)	
OG1RF Δ*bsrRS*	This study	
OG1RF Δ*croRS*	This study	
OG1RF Δ*liaFSR*	(67)	
OG1RF *trpX::Tn*	(46)	
OG1RF *metQ::Tn*	(46)	
OG1RF *10303::Tn*	(46)	
OG1RF *11614::Tn*	(46)	
OG1RF *12205::Tn*	(46)	
pP_23_::tdTomato	(61)	
OG1RF *bgsB::Tn* tdTomato	This study	
OG1RF Δ*bph* tdTomato	This study	
Chemicals		
Brain-heart infusion medium	Sigma-Aldrich	53286-100G
Spectinomycin	Chemie Brunschwig	J61820-06
Rat-tail collagen I	Corning	354236
Materials		
Transwells (upright imaging), 6.5 m, 0.4 µm membrane pores	Sarstedt	83.3932.041
Transwells (imaging in the chip), 6.5 m, 0.4 µm membrane pores	Corning	3470
Transwells used for CFUs	Corning HTS	CLS3381
Glass plate for imaging	Cellvis	P12-1.5H-N

### Generation of *E. faecalis* OG1RF ∆*bsrRS* and ∆*croRS* mutants

Deletion of the *bsrRS* operon (originally annotated as *yclRK*, but renamed here to *bsrRS* for consistency with nomenclature in *Enterococcus faecium* [60]) in the *E. faecalis* OG1RF background was carried out using the temperature-sensitive Gram-positive plasmid pGCP213 (68). To construct the deletion cassette, approximately 0.5 kb regions flanking the *bsrRS* coding sequence were PCR-amplified using primers listed in Table 2. Restriction sites for *SphI* and *EcoRI* were incorporated to facilitate cloning. The amplicons included the first and last ~15 amino acid codons of the gene to minimize unanticipated polar effects. These flanking regions were fused via overlap extension PCR to generate the final insert. Both the insert and pGCP213 plasmid were digested with *SphI* and *EcoRI* (New England Biolabs, USA) and ligated using T4 DNA ligase, following the manufacturer’s protocol (New England Biolabs, USA). The resulting plasmid, pGCP-yclRK, was transformed into competent *E. coli* Stellar cells, and positive transformants were screened using M13 primers and verified by PCR and Sanger sequencing. The construct was then introduced into electrocompetent *E. faecalis* OG1RF cells via electroporation, and mutant strains were isolated as previously described (68). Clones harboring the deletion were screened by PCR and Sanger sequencing using ycl screening primers.

**TABLE 2:** Primers^*a*^

Primer	Sequence	Application
yclRKdel1	5′CATGAGCATGCTGTTTGACATG3′	*bsrRS* deletion
yclRKdel2	5′GGAAATGAAATAATATCTGAGACGGATTCATTGTCTTCA3′	*bsrRS* deletion
yclRKdel3	5′TGAATCCGTCTCAGATATTATTTCATTTCCTGATGTTGAA3′	*bsrRS* deletion
yclRKdel4	5′TTGCGGAATTCTTATGGAATCATTTATTCT3′	*bsrRS* deletion
M13 F	5′GTAAAACGACGGCCAGT3′	*bsrRS* deletion
M13 R	5′CAGGAAACAGCTATGAC3′	*bsrRS* deletion
Screen ycl F	5′AGTAATAGCTTTCATTTTATCCATACCC3′	*bsrRS* deletion
Screen ycl R	5′ACAAGTACGTTAGTGATTGATGATATC3′	*bsrRS* deletion
pGCP213_F	AATTCCAGCACACTGGC	*croRS* deletion
pGCP213_R	AATTCTGCAGATATCCATCACAC	*croRS* deletion
CroSKO_up_F	GATATCTGCAGAATTATTAACTTTTAGCAAAATCTTCTAAATTTATCGTAA	*croRS* deletion
CroSKO_dn_F	TGGGGAGTTGGATATAAGATCGTTGACATCCCTGAACTTTTTCG	*croRS* deletion
CroSKO_up_R	CGAAAAAGTTCAGGGATGTCAACGATCTTATATCCAACTCCCCA	*croRS* deletion
CroSKO_dn_R	CAGTGTGCTGGAATTCACCACGTTCAAAATCTAGAATG	*croRS* deletion
croS_F	ATGCTCGTTAAACCTAAAAAGATTG	*croRS* deletion
croS_R	TTAACTCTCTGATTTCTTGTTGGTAC	*croRS* deletion
Tailed primer for Tnseq library prep.^*b*^	AATGATACGGCGACCACCGAGATCTACACTCTTTCCCTACACGACGCTCTTCCGATC tatgtatatctccggcgcgc	Nested PCR

^*a*^ Underlined bases correspond to restriction sites included to clone PCR products.

^*b*^ In tailed primer for Tnseq library prep., bold letters correspond to the Illumina P5 sequence; uppercase without bold font bases correspond to the TruSeq Read 1 primer binding site; lowercase bases correspond to transposon-specific binding sequence.

Generation of the *croRS* knockout mutant in *E. faecalis* OG1RF was also performed via allelic replacement using pGCP213, as described previously (68). The pGCP213 plasmid was first linearized by inverse PCR using primers pGCP213_F and pGCP213_R. Approximately 800 bp regions upstream and downstream of the *croS* gene were amplified from the OG1RF chromosome using primer pairs CroSKO_up_F/CroSKO_up_R and CroSKO_down_F/CroSKO_down_R, respectively. These flanking regions were then fused via overlap extension PCR using primers CroSKO_up_F and CroSKO_down_R. The linearized vector and fused insert were ligated using the In-Fusion HD Cloning Kit (Clontech, Takara, Japan) and transformed into *E. coli* Stellar competent cells. Correct constructs were confirmed by PCR and Sanger sequencing, and plasmids were extracted and introduced into *E. faecalis* OG1RF via electroporation.

Transformants were selected on erythromycin-containing media at 30°C, and chromosomal integrants were selected by growth at 42°C in the presence of erythromycin. To excise the integrated plasmid via homologous recombination, cultures were serially passaged at 37°C without antibiotic selection. Erythromycin-sensitive colonies were screened by PCR using primer pair croS_F/croS_R to confirm loss of the *croS* gene. Complete deletion of the *croS* gene, which overlaps with the downstream region of *croR*, was confirmed by whole-genome sequencing. Loss of expression of both CroR and CroS was validated by immunoblotting.

### Transformation of pP_23_::tdTtomato into *E. faecalis* OG1RF Δ*bph* and *bgsB::Tn*

Transformation procedures were previously described (69). In brief, 1 mL of overnight culture *E. faecalis* in 5 mL M17 medium (HIMEDIA cat. no. M1029-100G) was inoculated into 100 mL fresh SGM17 (M17 broth with 0.5M sucrose [HUBERLAB cat. no. A2211.1000] and 8% glycine [Fisher Scientific cat. no. BP381-1]) and incubated at 37°C for 18 h. Cells were pelleted at 1,000 × *g* for 10 min at 4°C. After discarding the supernatant, the pellet was washed with 2 mL ice-cold electroporation buffer (0.5 M sucrose with 10% glycerol [Sigma-Aldrich cat. no. G7893-1L], pH 7.0). Cells were pelleted at 1,000 × *g* for 10 min at 4°C. After repeating three washes, electrocompetent cells were resuspended in the electroporation buffer, divided into aliquots, and stored at −80°C. To perform electroporation, 1 µL of pP_23_::tdTomato DNA (1.0 µg/µL) was added to thawed electrocompetent cells. The cells were electroporated with 2.5 kV pulse, 25 µF capacitance, and 200 Ω resistance. Cells were recovered at 37°C for 2 h without aeration and plated on BHI agar plates.

### Culturing human colon organoids

Human colon organoids (donor #1, SCC357, 3d-GRO Merck human distal colon organoids; donor #2, SCC346, 3d-GRO Merck human distal colon organoids) were embedded in Matrigel (Corning cat. no. 356231) and grown into colonoids in a humidified incubator at 37°C and 5% CO_2_, using human intestinal Start medium. Grown colonoids were passaged as described previously (70, 71). Briefly, Matrigel domes were dissolved in ice-cold Basal medium, collected, and pelleted for 5 min at 200 × *g*, 4°C. The organoid pellet was resuspended by pipetting in 1 mL of ice-cold Basal medium, and an additional 9 mL of ice-cold Basal medium was added. After a second centrifugation step, when no more Matrigel was visible, colonoids were resuspended in freshly thawed Matrigel. A droplet containing 25 µL of the organoid suspension was added to a well of a 24-well plate (Corning cat. no. 3526), and the Matrigel domes were polymerized for 15 min at 37°C before 500 µL Start supplemented with 2 µM thiazovivin (Sigma-Aldrich cat. no. SML1045) was added. Organoids were passaged every 4–5 days at a ratio of 1:2 or 1:3 depending on the density. The composition of media is listed in Table 3.

**TABLE 3:** Organoid media

Compound	Source	Identifier	Basal medium	FMI Start	FMI Balance	FMI Balance supplemented
Advanced DMEM:F12	ThermoFisher	12634010	1×	1×	1×	1×
GlutaMax	ThermoFisher	35050061	1×	1×	1×	1×
HEPES, 1 M	ThermoFisher	15630106	10 mM	10 mM	10 mM	10 mM
B27 supplement	ThermoFisher	12587010		1×	1×	1×
N2 supplement	ThermoFisher	17502001		1×	1×	1×
N-acetyl-cysteine (NAC)	Sigma	A9165		1.25 mM	1.25 mM	1.25 mM
Human EGF	Sigma	E9644		50 ng/mL	5 ng/mL	5 ng/mL
Human RSPO1-Fc	Protein facility PTPSP	Custom		1 µg/mL	0.5 µg/mL	0.5 µg/mL
Human Noggin-Fc	SUN Bioscience	Custom		100 ng/mL	100 ng/mL	100 ng/mL
A83-01	Sigma	SML0788		0.5 µM		
Wnt Surrogate-Fc	ThermoFisher	PHG0401		0.5 nM	0.1 nM	0.1 nM
human [Leu15]-gastrin I	Sigma	G9145		25 nM	25 nM	25 nM
hNRG1	R&D	5898-NR-050		1 ng/mL	10 ng/mL	10 ng/mL
hIGF	Peprotech	100-11-1mg		100 ng/mL	100 ng/mL	100 ng/mL
hFGF	LuBioScience	100-18B		50 ng/mL	50 ng/mL	50 ng/mL
Thiazovivin	STEMCELL	72252		2 µM	2 µM	
DAPT	Sigma	D5942				5 µM
Vasoactive intestinal peptide (VIP)	Anawa Trading	AS-22872				330 ng/mL

### Culturing differentiated human colon organoids monolayers

To generate differentiated HCO in a Transwell (Sarstedt cat. no. 83.3932.041), organoids were collected by centrifugation (5 min, 200 g, 4°C), washed until all Matrigel was dissolved as described above, and broken down to single cells using 1 mL TrypLE (ThermoFisher cat. no. 12605010) for 7 min in a humidified incubator at 37°C with 5% CO_2_. Single cells were passed through a 40 µm cell strainer (pluriSelect cat. no. PS-43-10040-40), centrifuged (5 min, 200 g, 4°C), and resuspended in Start medium supplemented with 2 μM thiazovivin. Cells were then diluted and seeded onto Transwell membranes pre-coated with collagen (50 µg/mL rat tail collagen) at 100,000 cells in 100 µL to the 24-well Transwells and 45,000 in 50 µL for the 96-well Transwell plate. Five hundred microliters of fresh Balance medium was added to the basal side of the Transwell (200 µL to the 96-well plate wells). In the case of inverted Transwells (Merck cat. no. CLS3470) for high-resolution imaging, Transwells were transferred to a 6-well plate, and a custom-made PDMS ring was used to create a well around the basal side of the Transwell insert. 150,000 cells in 100 µL were added to the well in supplemented Start medium and left to settle for 24 h. The next day, the medium on the apical side of the regular Transwells was replaced with supplemented Balance medium and the inverted Transwells were returned to a regular configuration with 100 µL of supplemented Balance medium on the apical side and 500 µL on the basal side. Subsequently, the medium was changed every other day. When tissues reached full confluency (around 5 days), they were differentiated at the air-liquid interface (ALI) to accumulate mucus with FMI Balance supplemented medium at the basal side. On the third day at ALI, tissues were used for experiments involving bacterial coculture.

### Bacterial growth at the mucosal surface

All inoculations were performed with bacteria at stationary phase, washed in Hank’s balanced salt solution (HBSS) (ThermoFisher), and diluted to an OD_600_ of 0.5. One microliter of bacterial suspension (for 24-well plates), 0.4 µL (for 96-well plates), or 5 µL (for inverted Transwell) in HBSS was carefully added to the mucus-filled side and left to settle in the cell culture incubator for at least 30 min until use.

### Tn-seq for growth at the mucosal surface

The transposon library stock was diluted in BHI to an OD_600_ of 0.25 (3 mL) and grown to the deep stationary phase for 14 h. Starting this culture at a high OD_600_ was important to limit the number of generations and the consequent pre-selection before the experiment started (~4 generations for the bulk library), keeping a high transposon saturation in the inoculum. Then, cells were spun down, washed in HBSS buffer, resuspended in HBSS at an OD_600_ of 0.5 for use as the inoculum of the Transwell, and at an OD_600_ of 0.05 for the inoculum of the liquid cultures (BHI, cell medium [here Basal medium]). Six different 24-well Transwell inserts for donor SCC357 were infected using 1 µL, which is around ~250,000 *Ef* cells per insert. The infection progressed for 6 h, which resulted in ~6 generations without tissue damage by the library. Stationary-phase cells were used so that *Ef* were forced to adapt and grow exclusively from nutrients present in the mucosal environment. After infection, the tissue was homogenized using 100 µL of Triton X-100 (0.1% in PBS). Each insert was scraped until all the tissue was removed. Then, samples from all the inserts were pooled, vortexed, and pipetted vigorously, spun down (2 min, 8,500 rpm), washed in HBSS to remove traces of Triton X-100, and spun down again, and the pellets were stored at −80°C until subsequent processing.

In parallel, we prepared BHI and cell medium control samples, in which the inoculum used during the infections was grown in liquids for the same amount of duplication as the infections. For these, the inoculum was diluted in BHI and Basal medium (3 mL) to an OD_600_ of 0.02 for 3.5 h and 5 h, respectively. One milliliter of the culture was spun down, resuspended in HBSS, spun down, washed, and then stored at −80°C until subsequent processing.

The frozen pellets were processed using the QIAamp DNA Mini Kit (Qiagen). Samples were first thawed on ice for 20 min. Bacterial pellets were then resuspended in 200 µL ATL kit buffer, 30 µL 50 mg/mL lysozyme, and 20 µL proteinase K, mixed thoroughly by vortexing, and left shaking at 37°C for 30 min. After that, the steps in the kit were followed exactly. Genomic DNA concentration was measured using Nanodrop and Qubit. Library preparation and sequencing were performed at the Lausanne Genomic Technologies Facility at the University of Lausanne.

### Colony-forming units

To calculate CFU, liquid cultures were serially diluted in HBSS and plated on BHI plates. Transwell cultures were first resuspended in 0.1% Triton X-100 in HBSS (24-well Transwells in 100 µL and 96-well Transwells in 50 µL), as described above, then spun down, washed, and serially diluted in HBSS. The next day, colonies were calculated. CFU/mL were recorded, and doublings were calculated as log_2_ (final CFU per mL/initial CFU per mL).

### Confocal imaging of bacterial growth

For labeling mucus and MUC2 immunostaining (Fig. 1B), mucus was first labeled with Jacalin-biotin (5 mg/mL) with streptavidin-Cy5 (40 µg/mL in HBSS). The tissue was fixed with 4% paraformaldehyde in PBS for 30 min at room temperature on the apical side of the Transwell. The fixed tissue was washed three times with PBS, permeabilized with 0.2% Triton X-100 in PBS for 30 min at room temperature, and blocked with the blocking buffer (5% BSA and 0.01% Triton X-100 in PBS) overnight at 4°C. The tissue was then probed with the anti-MUC2 antibody in the staining buffer (2% BSA in PBS) overnight at 4°C. After washing three times with PBS, the goat anti-mouse IgG-Alexa Fluor 594 secondary antibody in the staining buffer was added to the apical side for one-hour incubation at room temperature. Labeled tissue was washed three times in PBS and stained with 300 nM DAPI for 10 min, followed by three PBS washes.

Inoculation of bacteria for imaging growth in mucus (for Fig. 1, 2 and 4; Fig. S1) was done as follows. *Ef* strains with plasmids encoding a fluorescent protein (WT expressing Dasher-GFP and *bgsB::Tn*, Δ*bph* expressing tdTomato) were grown in BHI broth (containing spectinomycin 120 µg/mL) from a single colony on a BHI plate with 120 µg/mL spectinomycin. Then, 500 µL of cells were spun down (2 min at 8,000 rpm), washed in HBSS twice, and resuspended in HBSS to an OD_600_ of 0.5. One microliter of the inoculum was used for a regular Transwell (Fig. 1, 4B and D) and 5 µL for the inverted Transwell (Fig. 2 and 4E; Fig. S1) (most of the unattached bacteria are then washed away in the chip). A 50:50 mixture was applied to the cases where WT and the mutant were grown together (Fig. 4D and E).

Epithelium labeling was achieved by incubating the Transwells with 5 µg/mL of CellMask Plasma Membrane Stain Deep Red diluted in the supplemented Balance medium for 30 min in the cell culture incubator at 37°C. The unbound stain was washed away with fresh supplemented Balance medium. Mucus labeling was performed using biotinylated Jacalin (5 mg/mL). A 500 µg/mL Jacalin solution in HBSS was applied at 1 µL on top of the Transwell or at 5 µL to the inverted Transwell. To label Jacalin-biotin, 40 µg/mL of streptavidin-Cy5 solution in HBSS-diluted bacterial culture was applied directly at the inoculation step (Fig. 2 and 4; Fig. S1).

Confocal imaging was performed using a Nikon Eclipse Ti2–E inverted microscope coupled with a Yokogawa CSU W2 confocal spinning disk unit and equipped with a Prime 95B sCMOS camera (Photometrics). Transwell inserts in the upright position (Fig. 1, 4B and D) were placed in custom-designed PDMS inserts, plasma-bonded to the glass in 12-well glass-bottom plates. Transwells were then imaged using a ×20 water immersion objective (Nikon, 0.95 NA) with Di01-T405/488/532/647 filters, 488, 561, and 640 nm laser lines, and 100 ms exposure of the fluorescent channels. Brightfield, GFP, Cy3, and Cy5 channels were collected. Images were collected as a z-stack of 2 or 3 µm steps. Fiji was used for the display and the analysis of images. To analyze the volume, image stacks were pre-processed using “Subtract background” function and “Gaussian Blur” filter on the bacteria channel, set at 1 µm, and then processed using 3D Objects counter plugin.

To image bacterium–mucus interactions at high resolution under dynamic flow conditions, inverted Transwell inserts were carefully inserted into the microfluidic chip as previously described (44). The chip featured three identical micro-milled open well channels with a glued glass coverslip (ref. 10812, ibidi GmbH, Martinsried, Germany) on the bottom. Metallic needles obtained from Luer-lock connectors (IP721-90; GONANO Dosiertechnik GmbH, Breitstetten, Austria) were then securely attached to the channel inlet and outlet ports. These needles were subsequently connected via Tygon-tube connectors (070534-08L-ND; ID 0.76 mm, wall 0.85 mm, Idex Health & Science GmbH, Wertheim, Germany) to polytetrafluoroethylene (PTFE) tubing (S1810-08; ID 0.5 mm, OD 1.0 mm, Bohlender GmbH, Grünsfeld, Germany). Additionally, a custom-designed 3D-printed lid with holes for the PTFE tubing, fabricated by Multi Jet Modeling on a Connex 500 printer (Objet) using VeroClear resin at the Additive Manufacturing Workshop (AFA) at EPFL, was positioned onto the Transwell inserts (basal compartment) to minimize evaporation of medium and supply fresh medium to the cells. Following the insertion, the microfluidic channels (apical compartment) were manually filled with a 5 mL Luer-lock syringe (Becton Dickinson cat. no. 309649) connected to the PTFE tubing via a metallic needle (GGA725050; GONANO Dosiertechnik GmbH, Breitstetten, Austria). One milliliter Luer-lock syringes (Becton Dickinson cat. no. 309628) with organoids medium were connected in a similar way through the lid. The syringes were operated via an external syringe pump system (Legato210, KD Scientific, cat. no. 78-8210), perfusing the apical compartment with medium indicated. In Fig. 2, organoid Basal medium is used, and in Fig. 4E and Fig. S1, a supplemented SILAC Advanced DMEM/F-12 Flex Medium (ThermoFisher) was used, containing 147.5 mg/L L-arginine hydrochloride (Apollo Scientific), 91.25 mg/L L-lysine hydrochloride (Apollo Scientific), GlutaMAX (ThermoFisher), 10 mM HEPES (ThermoFisher), and MEM NEAA solution (ThermoFisher). All experiments were performed at a flow rate of 5 µL/min and replenishing the basal compartment with organoid Balance medium at 0.5 µL/min. No phenol red color (the pH indicator dye in the Basal medium) was observed in the outlet when perfusing with SILAC medium (which is colorless), indicating that the barrier remained intact with no leakage. A total of five independent experiments, each with three Transwells, were selected and analyzed (two in rich medium and three in SILAC medium), and eight stable time-lapse imaged regions were selected for classification, four in each medium condition.

The chip was held in place in the stage-top temperature chamber (Okolab) at 37°C, with constant air/CO_2_ mix supply to support cell physiology. Transwells were then imaged using a ×40 water immersion objective (1.15 NA). Time-lapses were recorded for >18 h, every 30 min. To maintain the water for the objective, a custom-made water pump (a 30 mL syringe [Becton Dickinson cat. no. 301231] filled with water, using the same tubing connection and syringe pump described above) was used. The time indicated in all images is relative to the selected time point (*t* = 0) to enable tracking of the same position in the image stacks over time. Images were analyzed using Fiji. To calculate the gap size and distribution, we cropped individual large bacterial colonies and, for each image, calculated horizontal and vertical line profiles, averaged them, and calculated a moving average across all images.

### Tn-seq library sequencing

Library preparation and sequencing (performed at the Lausanne Genomic Technologies Facility, located at the University of Lausanne) procedures were previously described (28) with slight adjustments. Briefly, genomic DNA (500 ng) was initially sheared using a Covaris S220 with 450 bp insert settings (50 µL in microTUBES with AFA fiber; peak incident power: 175; duty factor: 5%; cycles per burst: 200; time: 50 s). Libraries were prepared with the xGen DNA MC UNI Library Prep Kit (IDT, protocol version v2) using xGen UDI-UMI adapters (IDT, 15 µM stock). With these adapters, P5 and P7 sequences are inverted compared to Illumina adapters, allowing transposon sequencing directly from read 1 (P5 side) in a single-end run. The purified ligated product was amplified by PCR with a primer specific for the Illumina P7 sequence (CAAGCAGAAGACGGCATACGA) and a second one specific for the transposon sequence (gcatcaccttcaccctctcc) with a 5′-biotin. PCR was performed with the KAPA HiFi HotStart ReadyMix kit (Roche). Cycling conditions were 98°C for 45 s, followed by 10 cycles of 98°C for 15 s, 60°C for 30 s, and 72°C for 30 s, with a final extension of 1 min at 72°C. The library was purified with SPRI beads at a 1× ratio.

The PCR product was captured with pre-equilibrated Dynabeads MyOne Streptavidin T1 (ThermoFisher). At least 1 mL of 1× B&W buffer (5 mM Tris-HCl pH 7.5, 0.5 mM EDTA, 1 M NaCl) was mixed with 25 µL of Dynabeads. After 1 min on a magnet, the supernatant was removed, and Dynabeads were washed with the same volume of 1× B&W buffer twice. The Dynabeads were resuspended with 50 µL of 2× B&W buffer (10 mM Tris–HCl, pH 7.5, 1 mM EDTA, 2 M NaCl). Then, 50 µL of the library was mixed with the washed Dynabeads, followed by a 30-minute incubation at RT on a rotator and placed on a magnet for 2 min. After discarding the supernatant, the Dynabeads were washed three times with 100 µL of 1× B&W buffer before the final elution in 40 µL H_2_O.

Half of the purified capture was used for the nested PCR with the Illumina P7 sequence (see sequence above) and a tailed primer made of the Illumina P5 sequence (boldface), the TruSeq Read 1 primer binding site (uppercase without boldface), and a transposon-specific binding sequence (lowercase): AATGATACGGCGACCACCGAGATCTACACTCTTTCCCTACACGACGCTCTTCCGATtatgtatatctccggcgcgc (see Table 2). The HiFi HotStart ReadyMix kit KAPA was used for nested PCR amplification, with the same cycling conditions as above (only nine cycles in this step). The final library was purified with SPRI beads at a 0.7× ratio. It was quantified with a fluorimetric method (QubIT, Thermo Scientific) and its size pattern analyzed with a Fragment Analyzer (Agilent).

Sequencing was performed on an Aviti (Element Biosciences) on a Cloudbreak Freestyle high-output flow cell for a 150-cycle single-end sequencing run. Clustering was performed with 1 nM library spiked with PhiX (Element Biosciences). Base calling and demultiplexing were done with bases2fastq (version: 1.7), and the data were further processed for transposon insertion analysis.

### Tn-seq data processing and analyses

Library sequencing analysis was performed at the Lausanne Genomic Technologies Facility, located at the University of Lausanne. Reads were trimmed for the Tn IR tag, Illumina adapter, and low-quality scores using cutadapt (v.4.8 parameters: -g "TATGTATATCTCCGGCGCGCCGCGACGCCATCTATGTGTCTAGAGACCGGGGACTTATCAGCCAACCTGTTA;min_overlap=70;max_error_rate=0.1" -A "GATCGGAAGAGCACACGTCTGAACTCCAGTCAC;min_overlap=10" -q 20 --pair-filter first). Only paired reads with a length larger than 50 nt for read 1 and 30 nt for read 2 were aligned against *Ef* strain OG1RF (accession number: CP002621.1) genome using BWA (v.0.7.18, parameters: mem -v 3 -T 0 -a -M). Alignment files were deduplicated using UMI_tools (v. 0.5.3, parameters: dedup—method=unique—paired --paired --umi-separator='c:'). The number of reads per insertion site was computed using a custom script that processes the genome read alignments from the BAM file (original or deduplicated). This script first filters the alignments using SAMtools (v 1.19) to select reads based on a specified flag (all alignments flag: -F 132; primary alignments flag: -F 388). It determines the exact position of the first base match on the reference genome by considering the read orientation and CIGAR string. The processed positions are subsequently analyzed and adjusted for any end clipping in the CIGAR string. Finally, it counts the occurrences of each insert site position in the genome and reports them in a sorted wiggle format file. In parallel, the number of read counts per gene locus was summarized with featureCounts (v.1.6.0, parameters: -p -s 0 -M --fraction).

Finally, primary and deduplicated sequences were analyzed in TRANSIT with the Tn5 “resampling” method within TRANSIT (GUI mode) to assess the conditional essentiality of genes between two conditions in three distinct comparisons. Specifically, we tested the “experimental sample” against the “control sample”: (i) mucus growth (5_muc) versus BHI control (3_bhi), (ii) mucus growth versus cell medium control (4_dmem), and (iii) cell medium growth versus BHI control. The initial library comparison to the inoculum is also available and shows minimal differences (1_start and 2_inn) (see Table S1).

### Enrichment analyses of functional processes using KEGG pathways and GO terms

For the classification of gene functions, a custom annotation (see Table S2, based on the previous version in reference 46) and the Kyoto Encyclopedia of Genes and Genomes (KEGG) database for the OG1RF strain were used. We selected 333 genes as significant (having log2FC < −2 in comparison to both the BHI and the cell medium). Gene ontology terms based on “GO_process” were applied from the NC_017316.1 full genome sequencing annotation in the GenBank and grouped accordingly using a custom-written R code, and the number of genes in each category is plotted in Fig. 3C.

### Growth curve measurement

Overnight-cultured *Ef* WT and mutants in BHI medium were spun down at 8,000 rpm for 2 min. The pellets were washed twice with PBS and diluted to OD_600_ = 0.02 in 100 µL of BHI medium or the minimal medium described above, with 1% (wt/vol) of D-galactose (Sigma-Aldrich), GalNAc (Sigma-Aldrich), GlcNAc (Adipogen), D-glucose (Carl ROTH), or D-mannose (Sigma-Aldrich), and loaded to a 96-well plate. The plate was sealed with parafilm and subjected to the SPECTROstar Nano plate reader (BMG LABTECH) for measuring OD_600_ every 10 min without shaking at 37°C.

### Statistical analysis

All statistical tests were run in Python. One-way analysis of variance (ANOVA) with post hoc Dunnett’s multiple comparison test was used, as described in the figure legends. We adjusted the *P*-values to be displayed as follows: *P* < 0.001, ***; *P* < 0.01, **; *P* < 0.05, *; and *P* ≥ 0.05, ns.

## Data Availability

Spreadsheets containing the source data used to generate the plots and the microscopy data displayed in all the figures are openly available via Zenodo at https://doi.org/10.5281/zenodo.17313938. The Tn-seq sequencing data have been submitted to the NCBI Sequence Read Archive under accession number PRJNA1353121.

## SUPPLEMENTAL MATERIAL

Supplemental figures msystems.01304-25-s0001.docx Fig. S1 to S5.

Captions msystems.01304-25-s0002.docx Captions for supplemental tables.

Table S1 msystems.01304-25-s0003.xlsx Pairwise comparison of Tn-seq conditions, including a list of enzymes classified with CAZy.

Table S2 msystems.01304-25-s0004.csv Full gene annotation for OG1RF.

ASM does not own the copyrights to Supplemental Material that may be linked to, or accessed through, an article. The authors have granted ASM a non-exclusive, world-wide license to publish the Supplemental Material files. Please contact the corresponding author directly for reuse.
