# Supplementary material for: Adaptation of Enterococcus faecalis to intestinal mucus revealed by a human colonic organoid model
Source: mSystems. 2026 Mar 10;11(4):e01304-25. doi: 10.1128/msystems.01304-25 (PMC13098192; doi:10.1128/msystems.01304-25)
Supplement: Supplemental figures — Fig. S1 to S5. [file msystems.01304-25-s0001.docx]

**Supplementary Figures**

**Adaptation of *Enterococcus faecalis* to intestinal mucus revealed by a human colonic organoid model**

Sofya Mikhaleva^1,2,7^, Po-Long Hsiao^1,2,7^, Amanzhol Kurmashev^3^, Caleb M. Anderson^4^, Cristina Colomer-Winter^4^, Julia A. Boos^3^, Pei Yi Choo^5^, Julia L.E. Willett^6^, Andreas Hierlemann^3^, Kimberly A. Kline^4,5^, Alexandre Persat^1,2^

1. Global Health Institute, School of Life Sciences, École Polytechnique Fédérale de Lausanne (EPFL), Lausanne, Switzerland
2. Institute of Bioengineering, School of Life Sciences, École Polytechnique Fédérale de Lausanne (EPFL), Lausanne, Switzerland
3. Department of Biosystems Science and Engineering, ETH Zürich, Basel, Switzerland
4. Department of Microbiology and Molecular Medicine, University of Geneva, Geneva, Switzerland
5. Singapore Centre for Environmental Life Sciences Engineering, Nanyang Technological University, Singapore, Singapore
6. Department of Microbiology & Immunology, University of Minnesota Medical School, Minneapolis, Minnesota, USA
7. These authors contributed equally to this work.

**
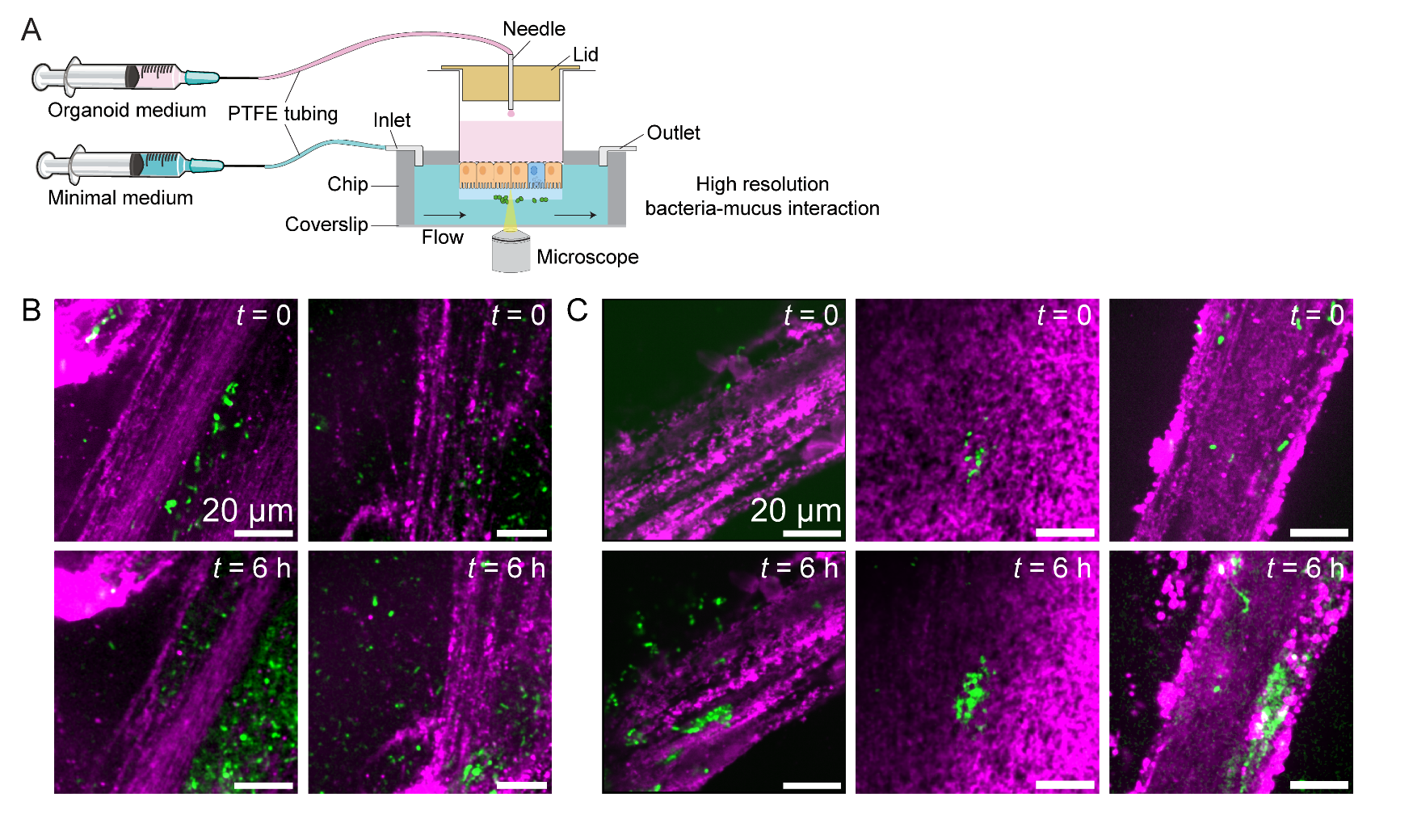
Figure S1. *Ef* grows in mucus under the flow of minimal medium.** (A) Schematic depicting the experimental setup using a Transwell-adapted microfluidic device for high-resolution confocal imaging. (B) Representative images of *Ef* colonies growing between thick mucus fibers and not forming biofilm-like colonies. (C) Representative images of *Ef* colonies growing inside the mucus but in areas with low mucus labeling between the mucus fibers. Images shown are acquired from three independent experiments.


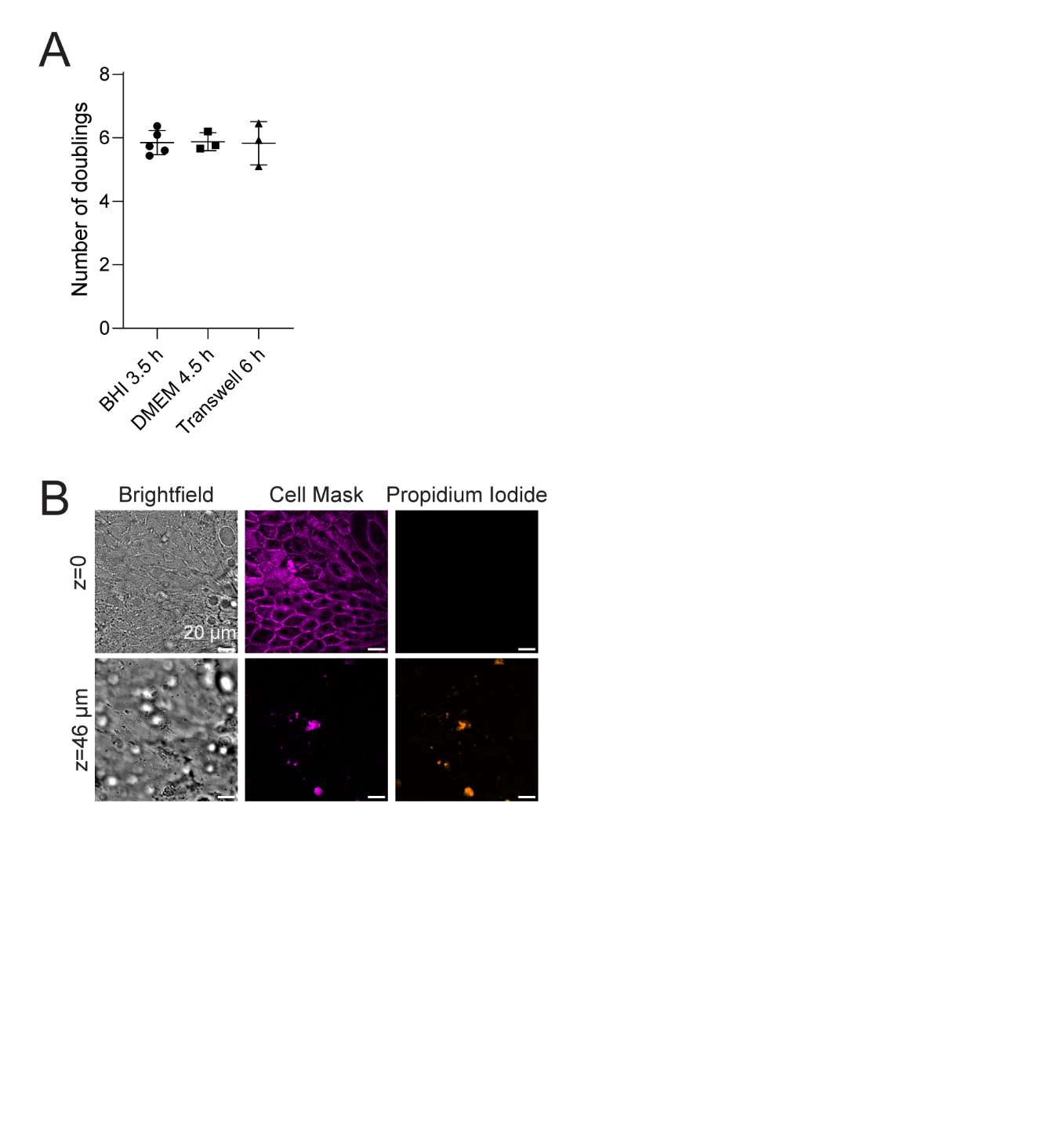


**Figure S2. Calibration of the Tn-seq condition.** (A) Incubation times of the library were calibrated to obtain similar doublings in rich medium (BHI), colonoid medium (DMEM), or Transwells. Each datapoint is a biological replicate. Mean and standard deviation are shown. (B) Epithelial cells do not show a significant loss of integrity after growing the library in mucus. The epithelial layer is stained with CellMask-DeepRed (magenta), and dead cells are labeled with propidium iodide (orange). Dead cells are only present in the mucus layer as in typical organoid cultures.


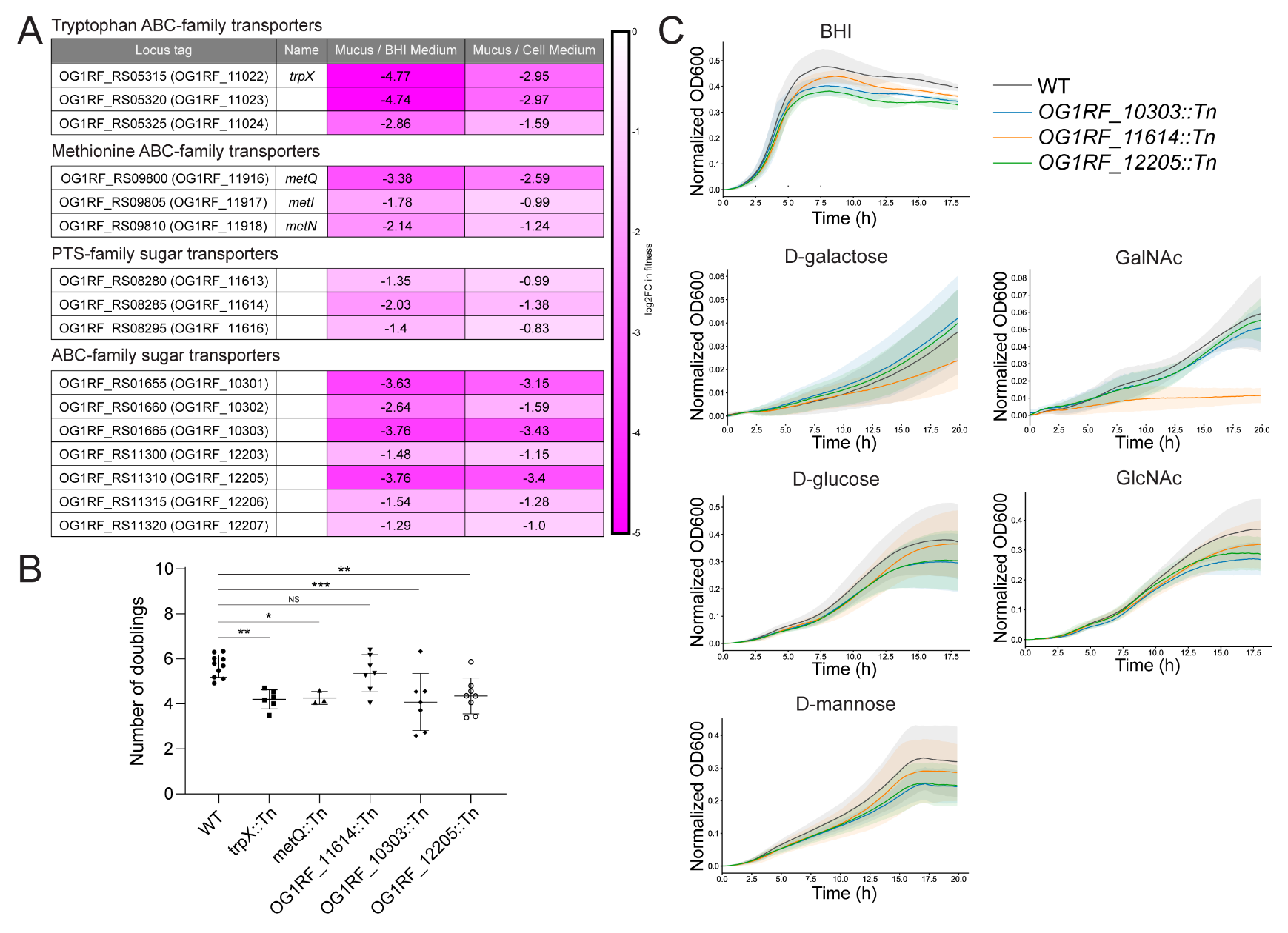
**Figure S3. The PTS and ABC sugar and amino acid transporting systems are required for *Ef* persistence in colonic mucus.** (A) Table of log2FC values calculated from the Tn-seq experiment for each selected gene among ABC- and PTS-transporters. (B) Colony forming units (CFU/ml) quantification of the mutants grown in mucus in comparison with the wild-type *E. faecalis* (at least 3 biological replicates for each mutant). Statistical significance was determined using one-way ANOVA followed by Dunnett’s multiple comparison test *(***, p<0.001, **, p<0.01, *, p<0.05, NS, p > 0.05).* Mean and standard deviation are shown. (C) Growth curves of transposon mutants and WT grown in BHI or liquid minimal medium supplemented with 1% indicated sugar in a 96-well plastic plate. Mean (line curves) and standard deviation (shade with corresponding color) are shown (biological triplicates).


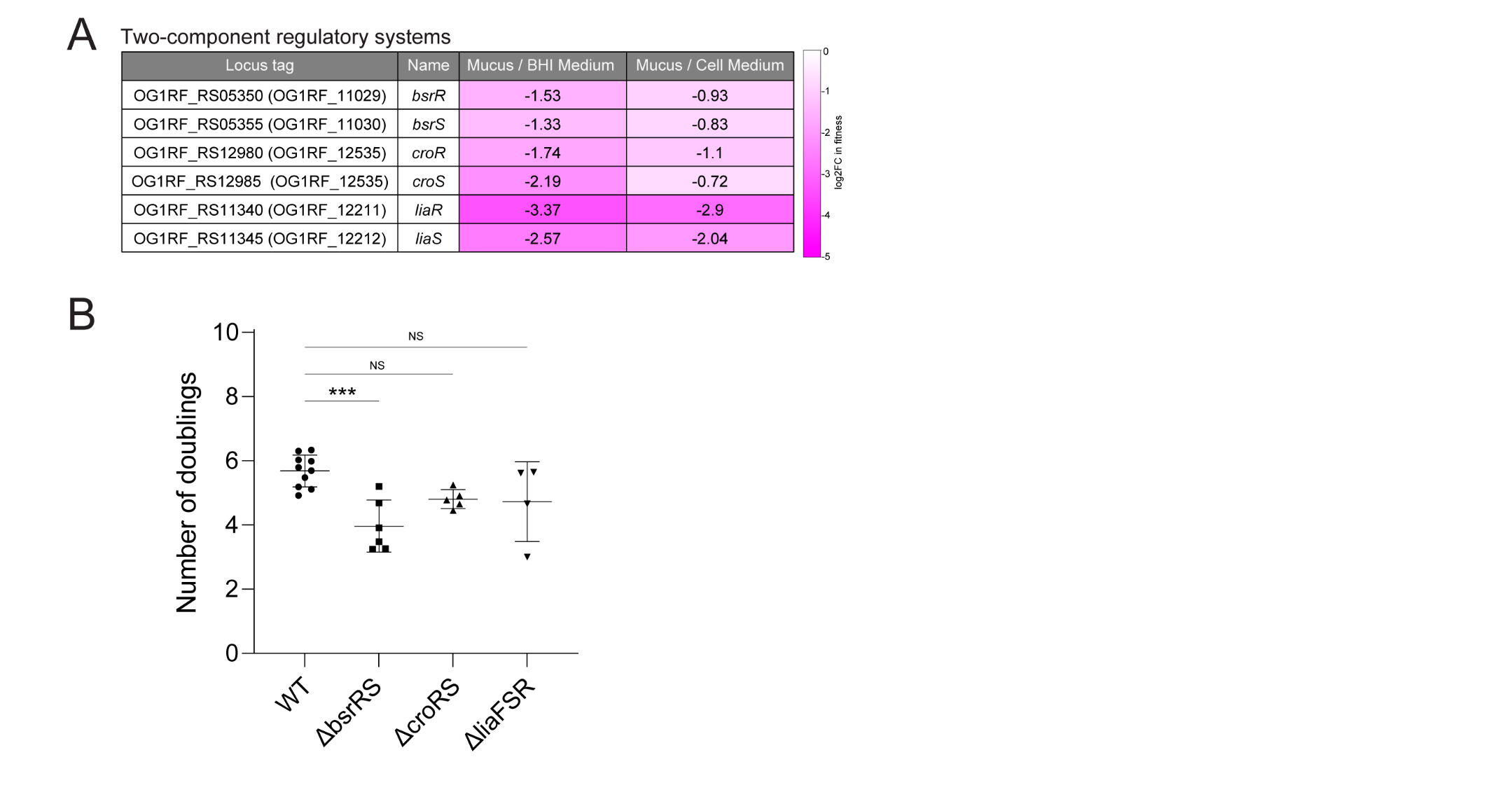


**Figure S4.** **The** **two-component regulatory systems are required for *Ef* persistence in colonic mucus.** (A) Table of log2FC values calculated from the Tn-seq experiment for each gene in the three selected two-component regulatory systems. (B) Colony forming units (CFU/ml) quantification of the deletion mutants grown in mucus in comparison with the wild-type *E. faecalis* (at least 3 biological replicates for each mutant). Statistical significance was determined using one-way ANOVA followed by Dunnett’s multiple comparison test *(***, p< 0.001; ns, p > 0.05).*


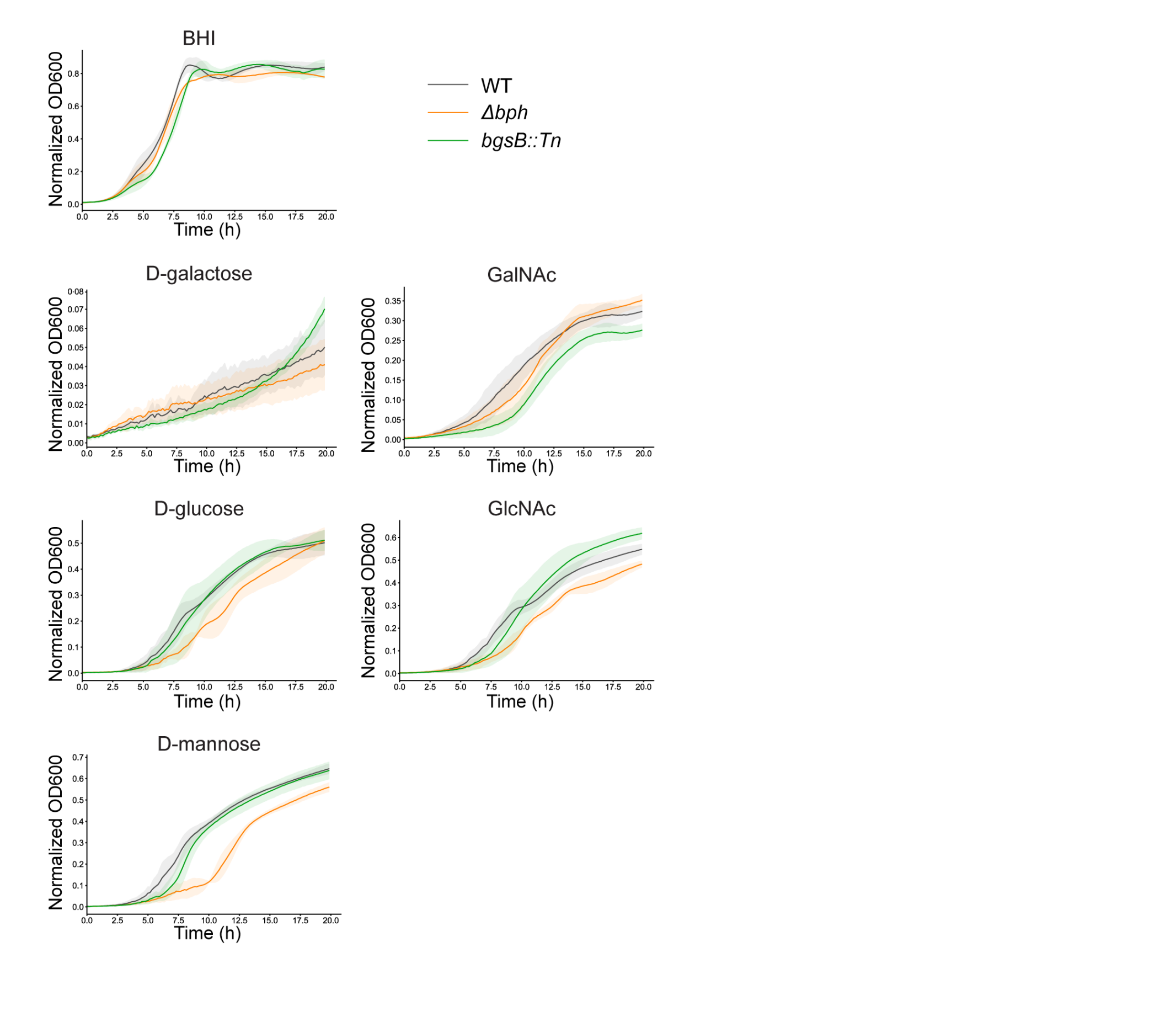


**Figure S5. *Δbph* mutant showed delayed exponential growth in mannose compared to WT.** Growth curves of *Δbph* and *bgsB::Tn* mutants and wild-type grown in BHI or liquid minimal medium supplemented with 1% indicated sugar in a 96-well plastic plate. Mean (line curves) and standard deviation (shade with corresponding color) are shown (biological triplicates).
