# Supplementary material for: Adaptation of Enterococcus faecalis to intestinal mucus revealed by a human colonic organoid model
Source: mSystems. 2026 Mar 10;11(4):e01304-25. doi: 10.1128/msystems.01304-25 (PMC13098192; doi:10.1128/msystems.01304-25)
Supplement: Captions — for supplemental tables. [file msystems.01304-25-s0002.docx]

**Legends for Supplementary Tables**

**Adaptation of *Enterococcus faecalis* to intestinal mucus revealed by a human colonic organoid model**

Sofya Mikhaleva^1,2,7^, Po-Long Hsiao^1,2,7^, Amanzhol Kurmashev^3^, Caleb M. Anderson^4^, Cristina Colomer-Winter^4^, Julia A. Boos^3^, Pei Yi Choo^5^, Julia L.E. Willett^6^, Andreas Hierlemann^3^, Kimberly A. Kline^4,5^, Alexandre Persat^1,2^

1. Global Health Institute, School of Life Sciences, École Polytechnique Fédérale de Lausanne (EPFL), Lausanne, Switzerland
2. Institute of Bioengineering, School of Life Sciences, École Polytechnique Fédérale de Lausanne (EPFL), Lausanne, Switzerland
3. Department of Biosystems Science and Engineering, ETH Zürich, Basel, Switzerland
4. Department of Microbiology and Molecular Medicine, University of Geneva, Geneva, Switzerland
5. Singapore Centre for Environmental Life Sciences Engineering, Nanyang Technological University, Singapore, Singapore
6. Department of Microbiology & Immunology, University of Minnesota Medical School, Minneapolis, Minnesota, USA
7. These authors contributed equally to this work.

**Table S1**. Pairwise comparison of Tn-seq conditions including a list of enzymes classified with CAZy.

**Table S2**. Full gene annotation for OG1RF.
